# Loss of cohesin deacetylation through KDAC8 inactivation leads to altered cohesin complex expression and chromosomal instability

**DOI:** 10.1371/journal.pone.0361705

**Published:** 2026-10-29

**Authors:** Tasha B. Toro, Razan H. Hammad, Thomas M. Huckaba, Terry J. Watt

**Affiliations:** 1 Department of Chemistry, Xavier University of Louisiana, New Orleans, Louisiana, United States of America; 2 Department of Biology, Xavier University of Louisiana, New Orleans, Louisiana, United States of America; Tulane University Health Sciences Center: Tulane University School of Medicine, UNITED STATES OF AMERICA

## Abstract

The cohesin complex is critical for both proper gene expression and for pairing sister chromatids during mitosis to ensure proper chromosome segregation. Acetylation and deacetylation of SMC3, a component of cohesin, is a critical regulatory mechanism controlling stability and reformation of the complex. Patients with Cornelia de Lange Syndrome have been observed to have mutations in cohesin-related genes, including in the lysine deacetylase KDAC8 (HDAC8), leading to disruption of complex formation and recycling. To further understand the cellular consequences of disrupting the SMC3 acetylation cycle, we utilized cell lines in which KDAC8 is replaced by a catalytically-inactive variant (8CDm) or knocked-out (8KO). Although the 8CDm cell line completes mitosis at the same rate as wild-type cells, 8CDm cells display severe chromosomal instability, characterized by increased presence of micronuclei and a significant subpopulation of cycling tetraploid cells. These defects were not observed in 8KO cells. Although total SMC3 protein levels are similar in all cell lines, SMC3 and other cohesin-associated genes are differentially expressed in the mutant cell lines. We hypothesize a model in which 8KO cells compensate for the inability to deacetylate SMC3 by increasing both SMC3 turnover and gene expression, and 8CDm mediated suppression of SMC3 turnover and expression results in fewer functional cohesin complexes and the severe chromosomal instability phenotype. Overall, these observations provide a greater understanding of the importance of SMC3 deacetylation by KDAC8 and insight into the phenotypic range observed in Cornelia de Lange Syndrome patients.

## Introduction

The importance of maintaining proper genome organization has long been appreciated; however, recent technological advances have revealed an intricate level of organization of the genetic material in the nucleus. This precise organization is essential for appropriate gene expression, efficient DNA repair, and keeping chromatids organized and appropriately paired during mitosis [1,2]. While many proteins are involved in organizing DNA, the cohesin complex performs critical functions during both interphase and mitosis. Cohesin is a multi-protein complex which assembles into a ring around two DNA strands, formed from SMC1A (UniprotKB Q14683), SMC3 (UniprotKB Q9UQE7), Rad21 (SCC1 homolog, UniprotKB O60216), and STAG1/2 (SCC3 homolog 1/2, UniprotKB Q8WVM7/ Q8N3U4). Many additional proteins are responsible for regulating cohesin function, such as loading the ring onto DNA and regulating the opening and closing of the ring, thereby influencing local DNA structure [3]. During interphase, the complex is responsible for maintaining the spatial organization of the chromosomes by tethering the base of DNA loops that are important for proper transcriptional regulation [4]. During S-phase, the complexes encompass sister chromatids to keep them together in mitosis until the metaphase-to-anaphase transition, when the complex is actively disassembled, allowing for proper segregation of sister chromatids to the daughter cells [4].

Several post-translational modifications modulate the behavior of the members of the cohesin complex. SMC3 undergoes an acetylation/deacetylation cycle which regulates its interactions with other proteins and chromatin (Fig 1) [5]. The acetylation of SMC3 is critical for the cohesin complex to perform sister chromatid cohesion, while deacetylation is required to allow an SMC3 molecule to be reused in a subsequent complex during interphase and during the next cell division cycle. SMC3 is acetylated at K105 and K106 (acSMC3) by the acetyltransferases ESCO1 (UniprotKB Q5FWF5) and ESCO2 (UniprotKB Q56NI9) after the complex forms around DNA [6–8]. Current evidence suggests that although ESCO1 is responsible for much of the SMC3 acetylation that occurs, ESCO2 is primarily responsible for acetylation during S-phase when cohesin is performing its role in sister chromatid cohesion [7]. Acetylation of SMC3 results in binding of CDCA5 (sororin, UniprotKB Q96FF9) to stabilize the cohesin-DNA complexes, which have a longer resident time while performing sister chromatid cohesion during S/G2 phases than while regulating genome organization and gene expression during interphase [7–11]. Release of acSMC3 from DNA occurs in a highly regulated process at the metaphase-to-anaphase transition [3]. At the centromere, this process involves displacement of SGO1 (shugoshin 1, UniprotKB Q5FBB7) from the cohesin complex and PTTG1 (securin, UniprotKB O95997) from ESPL1 (separase, UniprotKB Q14674), resulting in cleavage of Rad21 by ESPL1 [3,12–14]. An alternative cohesin release mechanism involving WAPL (UniprotKB Q7Z5K2) occurs along chromosome arms [3,11]. After cohesin disassembly, acSMC3 must be deacetylated by lysine deacetylase 8 (KDAC8 or HDAC8, UniprotKB Q9BY41) to release the N-terminus of Rad21 and to allow SMC3 to be recycled into subsequent cohesin complexes [5,15].

**Fig 1:**
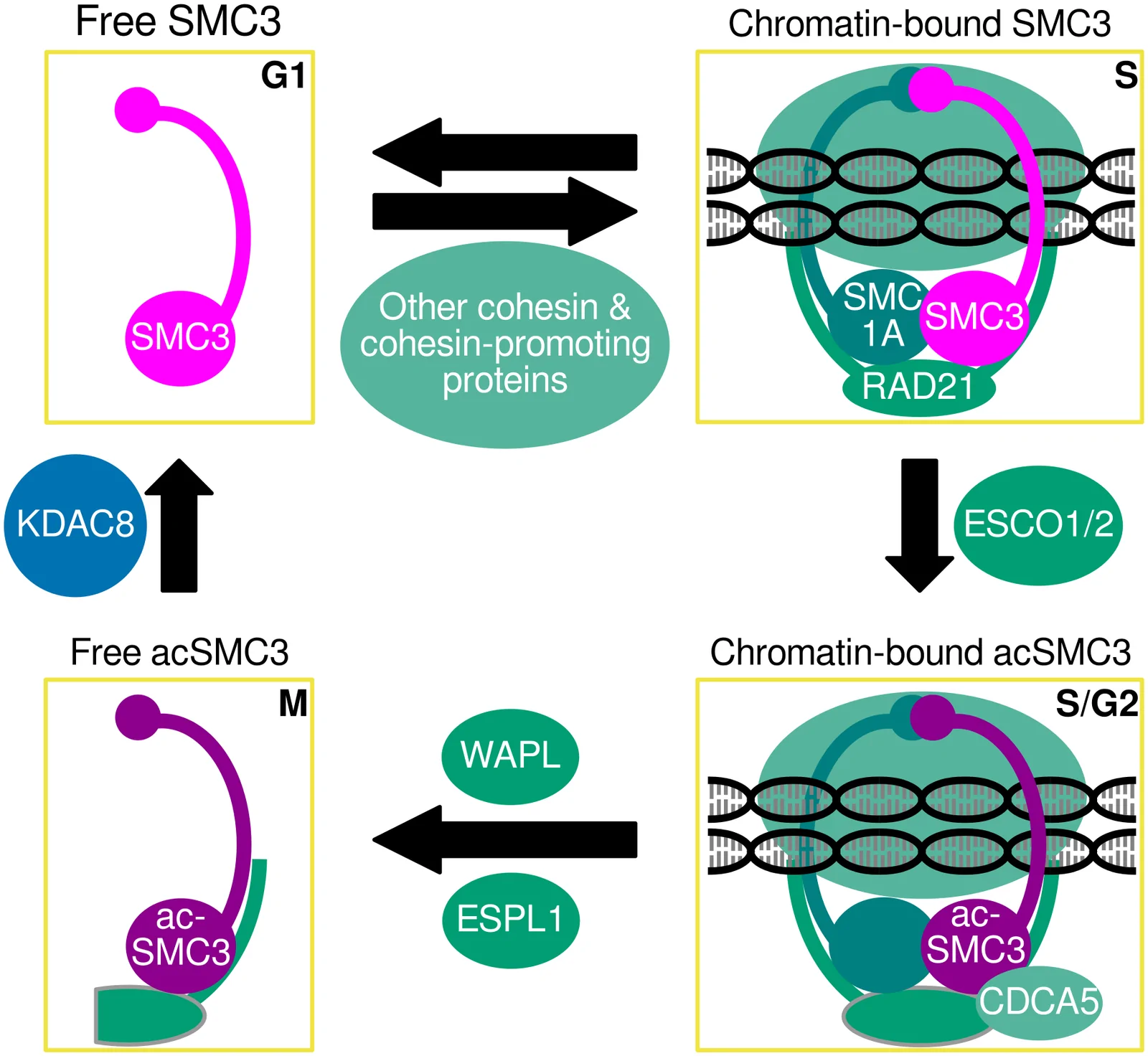
SMC3 acetylation/deacetylation cycle. Schematic diagram of SMC3 cycling in human cells. SMC3 moves between free and chromatin-bound states. A subpopulation of chromatin-bound SMC3 is acetylated by ESCO1 and/or ESCO2. After release from chromatin, KDAC8 must deacetylate free acSMC3 for it to be recycled into a new chromatin-bound complex. Chromatin-bound SMC3 functions in both gene regulation (during interphase) and sister chromatid cohesion (during S phase and mitosis). Acetylation of SMC3 appears most important for its sister chromatid cohesion function.

When cohesin is deficient in cells, such as when components of the complex are knocked down, defects in sister chromatid cohesion are observed in metaphase spreads. There is evidence that these cells develop aneuploidy, a hallmark of chromosomal instability (CIN), resulting from improper segregation driven by lack of cohesin [16]. CIN is a type of genomic instability characterized by changes in the number of whole or partial chromosomes and/or large rearrangements within chromosomes in a cell population. Sometimes, the entire genome is duplicated, which can lead to tetraploid cells containing two 2n nuclei as a result of cytokinesis failure. Alternatively, genome duplication can result in 4n DNA content in a single nucleus as a result of mitotic slippage such that the cells complete S-phase but do not successfully undergo mitosis [17]. CIN can also manifest as micronuclei, which are formed when genetic material does not segregate correctly and becomes encapsulated outside of the nucleus [18]. Once cells acquire one of these defects, they are more susceptible to accumulating additional defects if they continue to divide, leading to progressively more severe aberrations [19]. While deleterious for most cells, CIN is observed in the overwhelming majority of cancers, indicating that these cells can survive and even derive an adaptive advantage from the genomic instability [18,20].

In addition, dysfunctional cohesin leads to a group of disorders collectively known as cohesinopathies. The most common of these is Cornelia de Lange Syndrome (CdLS). This disease is characterized by a range of developmental defects, including facial anomalies and limb differences, as well as intellectual disability. While the majority of cases of the disease are associated with mutations in NIPBL (SCC2 homolog, UniprotKB Q6KC79), a protein responsible for loading the cohesin complex onto DNA, mutations in members of the cohesin complex and other associated proteins including SMC3 and KDAC8 have also led to CdLS in patients [21,22]. A few lines of evidence suggest that cohesin-driven gene expression defects may lead to CdLS; however, the extent to which gene expression and mitosis defects lead to CdLS and other related syndromes is not well established [4]. KDAC8 variants identified in CdLS patients are less active *in vitro* and lead to defects in the SMC3 acetylation cycle in cells [23]. In addition, knocking out KDAC8 in mice led to perinatal lethality with severe defects in skull formation, similar to those often seen in CdLS patients [24]. Recent work has documented that cells from CdLS patients with KDAC8 mutations accumulated chromosomal aberrations observed by karyotyping after many passages [25]. In addition, formation of micronuclei has recently been linked to altered acetylation of SMC3 driven by altered expression of ESCO1 and ESCO2 [26]. Taken together, these lines of evidence suggest a direct link between activity of KDAC8 and chromosome stability.

In trying to address the cellular role and functional importance of KDAC8, chemical inhibitors have often been employed; however, there are few inhibitors with reported specificity to KDAC8, and even those are likely not specific enough or potent enough to cleanly resolve the functions of KDAC8 due to observed off-target effects in cells [27–29]. To probe the cellular consequences of KDAC8 deacetylation while avoiding the issues associated with available inhibitors, we previously made and reported an engineered HT1080 (human fibrosarcoma derived from a male patient) cell line containing a missense mutation in the single genomic locus of the KDAC8 gene on the X chromosome, leading to endogenous expression of 8CDm (KDAC8H143A), which is catalytically inactive [30,31]. Initial characterization of this cell line led to novel insights into KDAC8 function. Transcriptome analysis of the 8CDm cell line compared to wild-type (WT) HT1080 cells identified more than eight thousand genes which were differentially expressed upon catalytic inactivation of KDAC8. These genes clustered around several cellular processes identified through a gene ontology (GO) analysis, including processes related to mitosis, DNA repair, transcription, and translation [31]. Cohesin-related proteins play a role in all of these processes, but it is not clear how, or even whether, the perturbations in all of these processes could be mediated solely by over-acetylation of SMC3, the only well-established consequence of losing KDAC8 function. While other substrates have been proposed for KDAC8, SMC3 is the only protein which has been clearly shown as a KDAC8 substrate and for which the function of the acetylation cycle is also known [32]. Here, we have further characterized the cellular consequences of catalytic inactivation of KDAC8, and report a complete KDAC8 knock-out derived from the same parent line. We report that the inactivation of KDAC8 led to severe chromosomal instability (CIN), which was largely absent from cells in which KDAC8 was not present (8KO). We also present a working model of how CIN arises in the presence of 8CDm but not when KDAC8 is absent.

## Materials and methods

### Cell growth and construction of KDAC8 knock-out cell line

All cell lines were cultured as previously described. Briefly, cells were cultured in growth media (minimal essential medium with Earle’s balanced salts and L-glutamine [MEM] supplemented with 10% fetal bovine serum, 50 U^-1^ Penicillin and 50 µg mL^-1^ Streptomycin) and incubated at 37°C with 5% CO_2_ [31].

HT1080 cells (HT1080 [HT1080] 9ATCC CCL-121) were obtained from ATCC. The 8CDm cell line was generated from HT1080 cells using CRISPR/Cas9 during a previous study [31]. The 8KO cell line was generated using a similar protocol targeting the KDAC8H143 locus. For this line, pSpCas9(BB)-2A-Puro (PX459) V2.0 containing the 8H143 specific gRNA sequence (CACCGTAGCAATTAACTGGTCTGGA) was transfected into HT1080 cells. Transformed cells were selected using 2.0 µg mL^-1^ puromycin. Cells were then plated in 96-well plates to achieve single colony dilutions. Candidates were genotyped by genomic DNA prep, PCR, and DNA sequencing of the PCR product to confirm the presence of a nonsense mutation using a protocol previously described in detail for the 8H143 locus [31]. The final 8KO cell line was prepared as a mixture of four individual isolates.

### Flow cytometry

Cells were grown to confluence under standard conditions. After trypsinization, cells were centrifuged at 500 xg for 5 minutes at 4°C. Cells were washed twice in ice-cold phosphate-buffered saline (PBS) and then resuspended in 500 µl ice-cold PBS. 4.5 mL ice-cold 70% ethanol was added while vortexing. Cells were stored at 4°C until further processing. Ethanol-fixed cells were centrifuged at 500 × g for 5 min, resuspended in 5 mL PBS, and incubated at room temperature for 10 minutes to equilibrate. Cells were then treated with 200 µg mL^-1^ RNase A at 37°C for 15 min protected from light and centrifuged at 500 × g for 5 min. Cells were resuspended in 5 µg mL^-1^ propidium iodide, incubated at 37°C for 30 min protected from light, and analyzed by flow cytometry using a FACSymphony A1 (BD Biosciences). Data were gated to eliminate double cells by front scatter height vs. area and then gated to eliminate debris by side scatter area vs. front scatter area. Fluorescence data was binned in steps of 500. Peaks were identified and fit to gaussian curves using scipy, with the areas of peaks assigned to 2n, 4n, and 8n populations, and the area between peaks as the intermediate populations. For population comparisons, residual data outside of the range from 2n to 8n was excluded.

### Fluorescence microscopy and immunofluorescence

Cells were grown under normal growth conditions on plasma-treated glass coverslips for two days and fixed in 4% formaldehyde for 15 min at room temperature. For analysis of the nuclei, after washing with PBS, cells were incubated with an Alexa Fluor^TM^ 488 phalloidin probe (Thermo Fisher) at a 1:500 dilution in PBS containing 5% goat serum for 1 hour at room temperature. After washing in PBS, coverslips were mounted using SlowFade^TM^ diamond antifade mountant with DAPI (ThermoFisher). Images were acquired using the same method described for immunofluorescence. Cells were visualized using a Nikon A1 microscope as described previously [29]. Using actin (phalloidin) signal to identify individual cell boundaries, DAPI signal was used to identify multinucleate cells and cells containing micronuclei. For each cell line, at least 1000 cells were analyzed, and the data are reported as the percentage of total analyzed cells containing either multiple nuclei or micronuclei. A Fisher exact test with Bonferroni correction was performed to compare mutant cell lines to WT. To measure nuclear area, the area of each clearly isolated nucleus was determined by counting the pixels with an intensity of at least 40% of the maximum signal intensity in sets of 16 images. At least 800 nuclei were measured for each cell line.

For SMC3 and acSMC3 immunofluorescence, cells were fixed, processed, imaged, and analyzed as described previously with a few modifications [29]. Primary antibodies listed below for immunoblotting were used at 1:50. Exposure times were always 100 ms for DAPI and 200 ms in other channels for each data set. For representative qualitative images, the intensity range of each fluorescence image channel was individually maximized over a linear range with no signal cropping, except for a uniform background subtraction to reduce non-specific haze, calibrated on either the intensity of an unpopulated area of the image (DAPI) or the intensity of control images with non-specific binding of secondary antibodies. To determine the ratio of acSMC3 to SMC3 signal, pixels belonging to individual nuclei were defined using the DAPI signal, and then the total signal in the other channels summed for those same pixels. The same background subtraction as for qualitative images was applied uniformly within each data set prior to calculating the ratio. Field images were captured in an unbiased manner from two slides prepared on separate dates for each cell line.

### Immunoblotting

For each sample, 60 µg total protein was loaded. The following primary, monocolonal antibodies were all used at 1:1000 dilution and incubated overnight at 4°C: KDAC8 (Cell Signaling Technologies 66042, RRID:AB_2895217), SMC3 (Cell Signaling Technologies 5696, RRID:AB_10705575), SMC3-acetylK105/K106 (Millipore Sigma MABE1073, RRID:AB_2877126), β-actin (Developmental Hybridoma Studies Bank JLA20, RRID:AB_528068). Appropriate secondary antibodies (Invitrogen 31464, RRID:AB_228378 or Invitrogen 31432, RRID:AB_228302) were used at 1:5000 and incubated for one hour at room temperature and detected using either Supersignal West Pico Plus Chemiluminescence substrate (Thermo Fisher) or Supersignal West Femto Maximum Sensitivity Substrate (Thermo Fisher). Detailed lysis and imaging procedures were previously reported and were followed as described there [31]. Immunoblot images were processed uniformly with a linear intensity range and for the maximum intensity range without exceeding saturation or cropping signal in any bands, except in long exposures in which the most intense bands are overexposed to show faint bands that otherwise would not be visible in the linear range.

### Cell growth rate analysis

Cells were plated at 0.65x10^5^, 0.32x10^5^, 0.16 x10^5^, and 0.08 x10^5^ cells per well in a 12-well tissue culture plate in triplicate for each cell line. Cells were maintained under normal growth conditions. After 24 hours, cells plated at the highest density were trypsinized, harvested, and centrifuged for 1 minute at maximum speed. Cells were resuspended in 50 µl media, mixed in a 1:1 ratio (v/v) with 0.4% trypan blue (Life technologies), and counted in duplicate using a Countess II cell counter (Life Technologies). Duplicate counts were averaged to produce a live cell count per mL for each well. For 3 subsequent days, the highest density cells were harvested each day and counted as described. From the raw cell counts, average cells per well was calculated for each well. The average cell counts for each well and each condition were used in subsequent calculations. Fold increase in cell density was calculated as cell density at harvest divided by the starting density for each condition. The log_2_ of the fold increase was divided by the number of days of growth to get the number of cell doublings day^-1^. Reported doublings day^-1^ represents the average across all starting densities.

### Live cell imaging

Approximately 4000 cells per well in 200 µL growth media were plated in an Incucyte Imagelock 96-well microplate (Sartorius). Phase images at 10X magnification were taken in ImageLock mode every five minutes for 36 hours using an Incucyte S3 live cell analysis instrument (Sartorius). Time-lapse images for each field were opened as a stack in ImageJ and the MTrackJ plugin was used to mark the progression of division of individual cells [33]. The following events were recorded for each tracked cell division: start of mitosis (identified as rounding up), start of cytokinesis (identified as formation of a midbody structure), and end of cytokinesis (abscission resulting in two distinct daughter cells). For each cell line, at least 60 mitotic events were tracked and the time between recorded events was calculated based on the number of frames between events. Mitosis time represents the time from the start of mitosis to the start of cytokinesis, while cytokinesis time represents the start of cytokinesis to the end of cytokinesis. The average time and standard deviation for each process for each cell line was calculated. Representative images were uniformly contrast enhanced without signal cropping, and zooms were performed without interpolation from the original 10X magnification.

### 8KO gene expression analysis

RNA-seq analysis and data processing for the 8KO line was performed as previously described for the 8CDm line, except using a false discovery rate adjustment of α = 0.001 [31]. Gene Ontology (GO) analysis was performed as previously described, but using the basic ontology set 2025-06-01 and GO human annotation set 2025-06-01 [31].

### Statistical analysis

Most data sets were compared using a two-sided Mann-Whitney U test to minimize effects of non-normal distributions and outliers, and also evaluated for effect size using Cohen’s d. Significance values are reported as the adjusted p value after applying the Bonferroni correction for multiple testing of pairwise comparisons. Differences were considered significant if both p (adjusted) < 0.05 and Cohen’s d magnitude ≥ 0.3. A two-sided Fisher exact test was used to identify differences in counted samples (binucleate cells and micronuclei), for p (adjusted) < 0.05. For all tests, n.s. indicates non-significant comparisons. Sample size, mean, standard deviation, median, test statistics, and unadjusted p values for every data set tested for significance and flow cytometry data are listed in S1 Table.

## Results

### Nuclear acetyl-SMC3:SMC3 ratios are similar in KDAC8-inactivated and KDAC8-knockout cells

To determine whether we would see similar phenotypes in cells in which KDAC8 was completely knocked-out versus inactivated, we created an 8KO line using a CRISPR/Cas9 based method similar to the one used to create 8CDm in HT1080 cells to avoid introducing additional variability into the experiment [31]. The 8KO line was initially verified by sequencing (S1 Fig), and allowed to grow for nearly three months, inclusive of the duration of the selection process, to match the age that the 8CDm line previously reached after genome editing. Both lines therefore model a long-term adapted state rather than being indicative of an acute disruption of protein function. In contrast to the WT and 8CDm lines, we could not detect any KDAC8 protein expression in the 8KO line even after a long exposure, and, as expected, we observed an increase in acSMC3, even though the total SMC3 levels were not elevated in the mutant cell lines (Figs 2A and S2). The increase in acSMC3 in the nucleus of both 8CDm and 8KO cells was clearly observed in fixed cells by immunofluorescence (Fig 2B), with both lines having a ratio of acetylated SMC3 to total SMC3 about 100-fold greater than observed in WT cells (Fig 2C).

**Fig 2:**
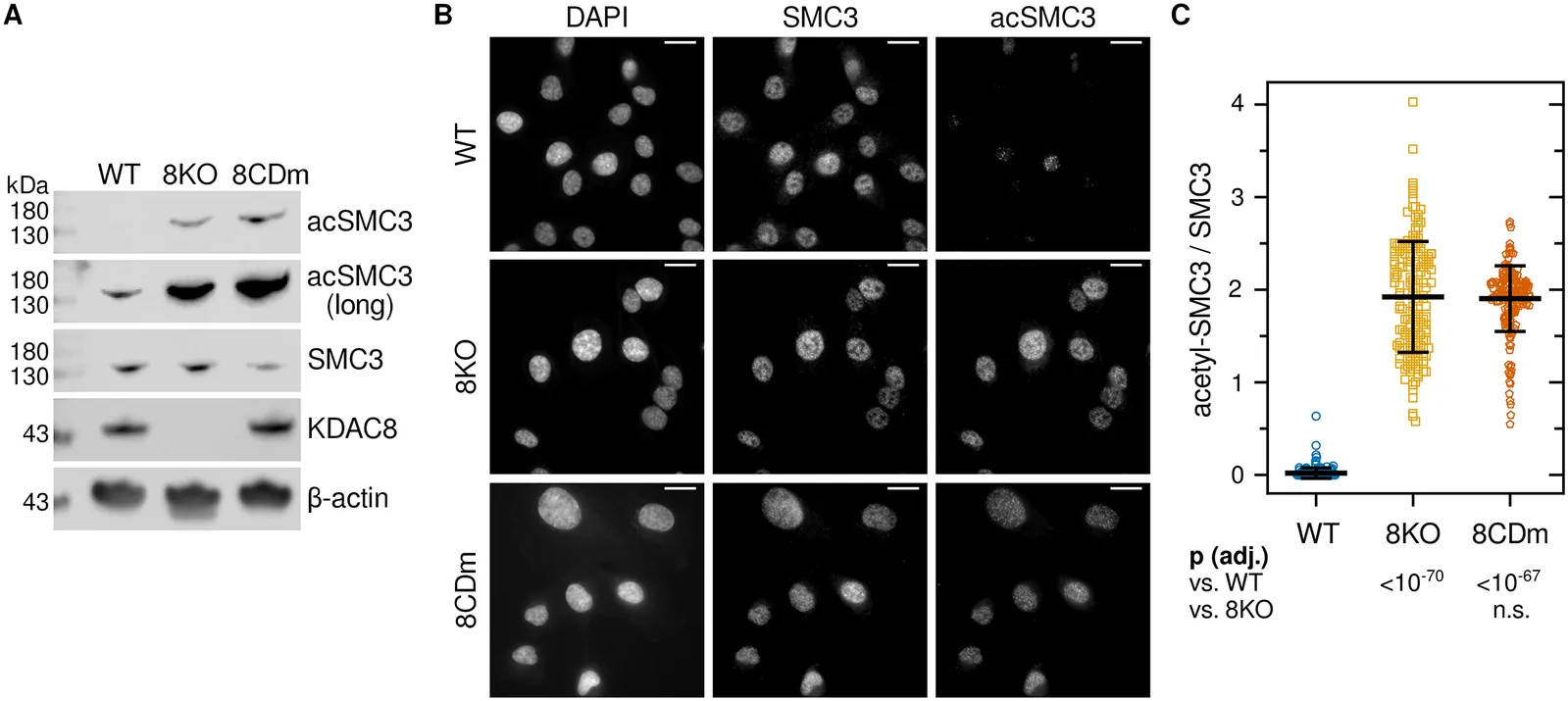
8CDm and 8KO cells accumulate nuclear acSMC3. **(A)** Representative immunoblots of lysates showing acSMC3 and total SMC levels in each cell line. A long exposure of acSMC3 is shown to allow comparison of weak and strong bands. KDAC8 presence (WT, 8CDm) or absence (8KO) is also shown. β-actin serves as a loading control. **(B)** Representative immunofluorescence of SMC3 and acSMC3 (SMC3 acK105 acK106) in fixed cells is shown for each cell line. Nuclei are marked with DAPI stain. The scale bar represents 20 µm. **(C)** The ratio of total acSMC3 to total SMC3 signal in the nuclei of individual cells (n ≥ 189 nuclei per cell line). Bars represent mean and standard deviation, and p values were determined by Mann-Whitney U tests.

### Genetic inactivation of KDAC8 catalytic activity leads to enhanced chromosomal instability

Previous characterization of the 8CDm cell line, in which catalytic activity of KDAC8 was genetically inactivated, revealed that gene expression was changed compared to wild-type (WT) for several cellular processes, including downregulation of genes involved in several processes related to mitosis and cytokinesis [31]. We used fixed DAPI-stained cells to determine whether genetic inactivation of KDAC8 catalytic activity led to an increase in multinucleate cells or other hallmarks of CIN (Fig 3A). As HT1080 cells are an immortal cell line originally isolated from a fibrosarcoma, a low level of multinucleate cells, as well as other markers of CIN, were present in WT cells; however, we saw a greater than 5-fold increase in the number of multinucleate cells in the 8CDm cell line compared to WT (Fig 3B). Almost all of these were binucleate, but we did observe a few cells with more than two nuclei in the 8CDm cell line; the most extreme example contained at least 8 nuclei observable by DAPI. We also observed micronuclei (Fig 3A). Similar to the increase in multinucleate cells, 8CDm cells had a greater than 6-fold increase in the percentage of cells containing micronuclei compared to WT cells, and many of these cells contained multiple micronuclei, sometimes in conjunction with multiple nuclei or very large nuclei (Fig 3A and 3C). In addition, the nuclear area of the 8CDm cells often appeared larger and more variable in area than in the other cell lines (Fig 2B). We quantified this observation by measuring the DAPI area in the same fixed cells and found the nuclei of the 8CDm cells were more heterogenous in size than WT cells, and the median nucleus area for the 8CDm cells was approximately 60% larger than WT cells (Fig 3D).

**Fig 3:**
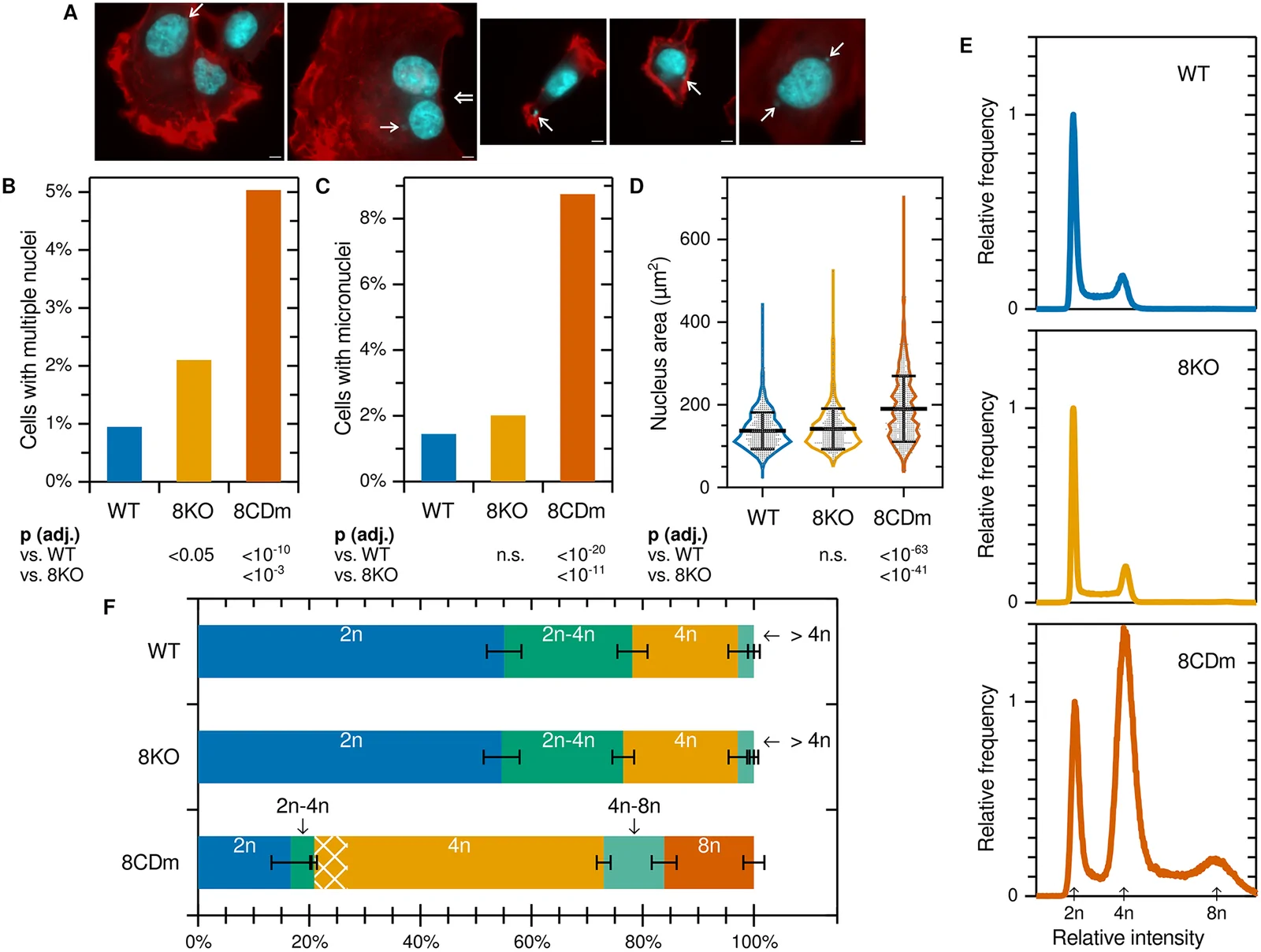
8CDm cells show enhanced chromosomal instability. **(A)** Representative images showing examples of nuclear anomalies in the 8CDm cell line. Images were obtained by fluorescent microscopy of fixed cells labelled with DAPI (DNA; cyan) and phalloidin (f-actin; red). The full set of images from this data set were used for the analysis in subsequent panels. Scale bar represents 10 µm. Single line arrows indicate micronuclei. Double line arrow indicates binucleate cell. **(B)** Cells containing more than one nucleus were counted using the fixed, DAPI stained cells (n ≥ 1000 cells analyzed per cell line). p-values were determined by Fisher exact tests. **(C)** Cells containing micronuclei were counted using the fixed, DAPI stained cells (n ≥ 1000 cells analyzed per cell line). p-values were determined by Fisher exact tests. **(D)** Nucleus area of cells was determined by measuring the DAPI signal. Measurements of individual nuclei of each cell line are plotted (n ≥ 800 nuclei per cell line). Bars represent mean and standard deviation, and p values were determined by Mann-Whitney U tests. **(E)** Representative raw traces of flow cytometry for WT (top; blue), 8KO (middle; yellow), and 8CDm (bottom; red) cell lines (n ≥ 400,000 total events for each trace). DNA content in each peak is indicated on bottom with arrow. 2n peak height was set to 1 for each cell line to compare relative frequencies within each line. The 2n peak corresponds to diploid cells in G1. **(F)** The percentage of the total cell population in and between each peak for pooled flow cytometry data (≥ 3 independent experiments for each cell line; n ≥ 1,700,000 total events for each cell line). DNA content is labeled for each bar section. Error bars represent standard error of the mean. The hashed yellow region in the 8CDm line represents the estimated fraction of the 4n cells that would correspond to G2/M cells, based on the number of 2n cells.

Strikingly, the 8KO line did not display the severe CIN observed in the 8CDm line (Fig 3). There was a slight increase in multinucleate-containing cells in the 8KO line compared to WT; however, this phenotype was much less pronounced than in the 8CDm line (Fig 3B). Unlike 8CDm cells, 8KO cells did not have a statistically significant change in micronuclei frequency nor in nuclear area (Fig 3C and 3D). Together, these data indicate that catalytically-inactive KDAC8 is causing severe CIN that is not observed when KDAC8 is absent.

The variation in nuclear area suggested that the nuclei of the 8CDm cells contained more DNA, on average, than WT cells, and that there might be more cell-to-cell variability than expected for cells with uniform ploidy. We used flow cytometry to determine whether inactivation of KDAC8 catalytic activity led to changes in the cell cycle profile and/or DNA content. HT1080 WT cells produced a typical profile with a G1 (2n DNA content) peak and a second smaller G2/M peak (4n DNA content), representing cells that have completed S-phase but have not yet completed cell division (Fig 3E). The flow cytometry profile for 8KO cells was indistinguishable from WT cells (Fig 3E and 3F). Surprisingly, the profile for 8CDm cells was substantially different from both the WT and 8KO cells. 8CDm cells resolved into three distinct peaks, which corresponded to 2n, 4n, and 8n DNA content (Fig 3E and 3F). Here, the largest peak was the 4n peak. The number of cells in that peak far exceeded the number expected based on the size of the 2n peak and the ratio of approximately 3:1 of 2n to 4n cells in WT. Therefore, we concluded that only a small portion of the 4n cells were likely to be diploid cells in G2/M (represented by the white hatch marks in Fig 3F). The tetraploid population includes the increased number of binucleate cells observed in 8CDm (Fig 3B), but the increase in these cells is insufficient to account for most of the expanded 4n population. Thus, the majority of the 8CDm tetraploid cells must be mononucleate cells in G1. We also observed an approximately 3:1 ratio between the additional 4n cells and the 8n population, suggesting that the 8n population was primarily tetraploid cells in G2/M. We also used scatter measurements from the flow cytometry data to assess the relative sizes of the cells in each population. Consistent with continued cycling of the 8n population, the volume distribution of the 2n and 4n 8CDm cells is indistinguishable from WT or 8KO cells, and the 8n population retains the same distribution shape but shifted to a significantly larger volume (S3 Fig). The population of cells between the 4n and 8n peaks, then, mostly correspond to tetraploid cells undergoing DNA synthesis, likely mixed with a small population of other aneuploid cells. Altogether, the flow cytometry suggests that the majority of the 4n 8CDm cells, despite a genome duplication event, were still actively cycling. These data were consistent with the larger, more heterogeneous nuclei observed in the 8CDm cells, suggesting that that the observed variation in area is due to the fact that many 8CDm cells contained greater than 2n DNA content. Thus, the 8CDm line displayed a severe CIN phenotype, harboring several hallmarks of CIN, which is expected for aneuploid cells that lose the ability to maintain genomic integrity [19].

### Cells with inactive KDAC8 do not have altered cell division timing despite chromosomal instability

The strong CIN phenotype and aberrant flow cytometry profile observed in the 8CDm cell line suggested the potential for a problem during cell division. To test this, we used live cell imaging to track the timing of individual mitotic events. We measured how long cells took to undergo mitosis, defined as the period from cell rounding to the formation of a visible midbody connecting the two new cell bodies. All three cell lines completed mitosis in the same amount of time, indicating that any abnormalities occurring during mitosis did not affect the speed at which cells performed mitosis (Fig 4A). Similarly, the three cell lines were indistinguishable from each other in time spent completing cytokinesis, defined as the time from formation of a midbody to the end of cytokinesis (Fig 4B). Although the 8KO and 8CDm lines had more cytokinesis events of particularly long duration, those cells did complete cytokinesis and did not rise to the level of statistical significance. We did not observe cells failing to complete cytokinesis and giving rise to multinucleate cells in the samples for any of the cell lines, indicating that the increase in multinucleate cells (Fig 3B) is probably not primarily due to a significant increase in cytokinesis failure events. There were no distinct differences in visual appearance of mitosis or cytokinesis between any of the cell lines (S4 Fig). Therefore, we concluded that the CIN defects in 8CDm are not primarily arising due to disruptions of cell division, despite previous observations that cellular processes related directly to both mitosis and cytokinesis were downregulated in the 8CDm line [31].

**Fig 4:**
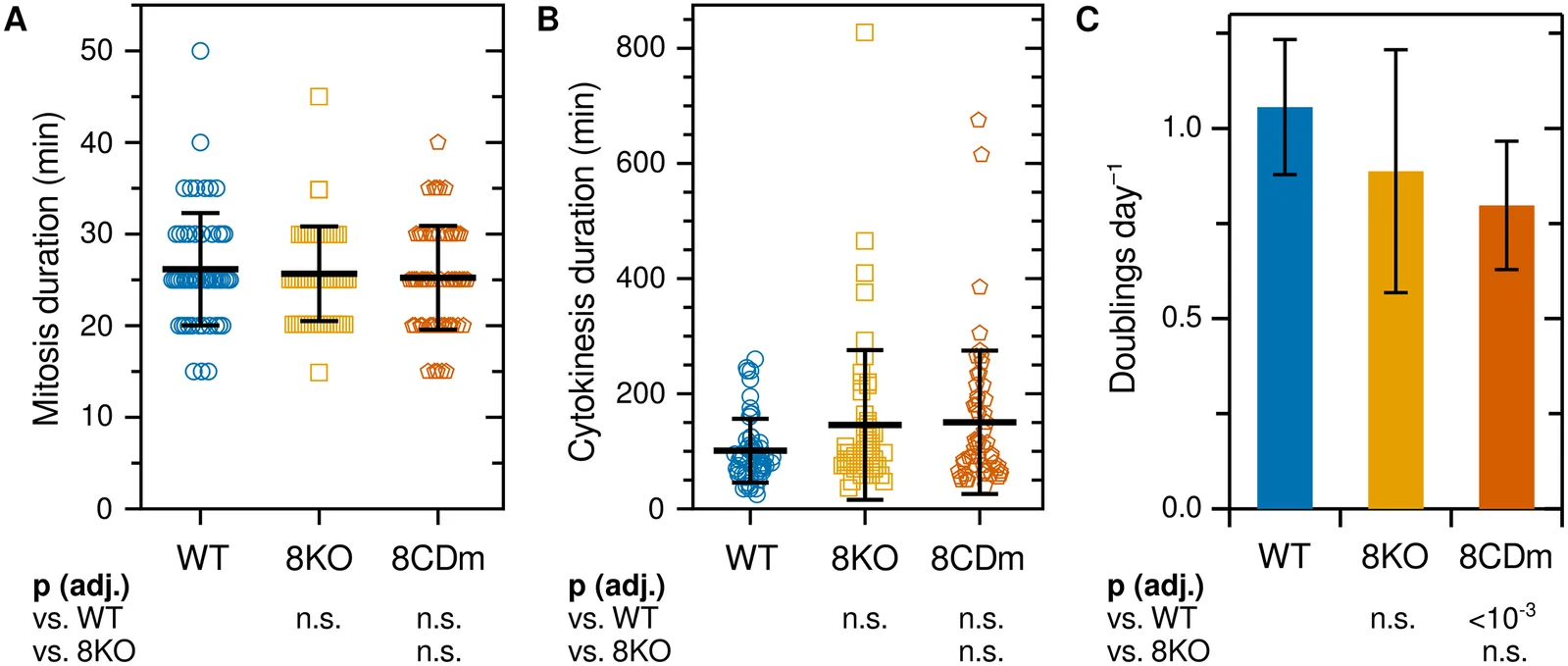
Loss of KDAC8 activity does not disrupt cell cycle timing. **(A)** Time-lapse images from live cell imaging were used to determine the length of time between rounding up and midbody formation of individual mitotic cells (n ≥ 60 mitotic events per cell line). Each cell is plotted, and mean and standard deviation for each cell line are shown as a bar and whiskers. There were no significant differences between any of the cell lines (p < 0.05, Mann-Whitney U test). **(B)** Time-lapse images from live cell imaging were used to determine the total cell division time, defined from rounding up to formation of two distinct daughter cells (n ≥ 60 mitotic events per cell line). Each cell is plotted, and mean and standard deviation for each cell line are shown as a bar and whiskers. There were no significant differences between any of the cell lines (p < 0.05, Mann-Whitney U test). **(C)** Each cell line was plated in triplicate at known densities and allowed to grow for a set period of time before harvesting and counting cells at 4 timepoints. Average doublings day^-1^ was calculated for each cell line from the raw cell counts. Bars represent standard deviation, and p values were determined by Mann-Whitney U tests.

To further probe the nature of the CIN defect in the 8CDm cell line, we monitored cell growth over five days by plating cells at fixed density and counting cells at fixed timepoints. Using these data, we calculated doublings per day for each cell line. Only the 8CDm line showed a significant reduction in doubling time compared to WT cells (Fig 4C). As we did not observe a measurable population of potentially apoptotic cells in flow cytometry with less than 2n DNA (Fig 3E), we hypothesize that a small fraction of the 8CDm cells are no longer dividing, or not dividing as frequently as the other cells in the 8CDm cell line population, even after undergoing DNA synthesis. However, the majority of the cells containing 4n DNA must have been actively cycling and growing, as a much greater reduction in doublings per day would have been expected if only the one-third of the population with a 2n DNA content was cycling normally.

### Catalytic inactivation or knockout of KDAC8 result in distinct expression changes of cohesin-related genes

We previously reported results of GO analysis of the 8CDm cell line, which revealed the downregulation of genes involved in several cellular processes related directly to both mitosis and cytokinesis, including “mitotic sister chromatid segregation” (GO: 0000070) [31]. Based on the similar results we obtained when we analyzed cell division in the two mutant cell lines, we performed the analogous gene expression and GO analysis of the 8KO line to determine whether complete loss of KDAC8 would result in similar gene expression changes. Despite a similar overall number of significant gene expression changes in the 8KO line compared to the 8CDm line (S2 Table), we discovered that we could only correlate the 8KO expression changes to a single process related to the cell cycle through GO analysis: upregulation of genes involved in “regulation of cell cycle” (GO:0051726) (S3 Table), which was associated with downregulated genes in 8CDm cells [31]. The much larger number of GO terms related to cell division processes associated with the gene expression changes in the 8CDm line compared to the 8KO line is consistent with the larger number of chromosomal anomalies the 8CDm cell line compared to the 8KO line. However, the lack of apparent differences in mitosis and cytokinesis timing (Fig 4) suggested that GO analysis alone was insufficient to account for the CIN defects.

Therefore, we next looked specifically at gene expression changes in cohesin-related genes. First, we examined mRNA expression of the proteins forming the cohesin complex. Based on our observation that total SMC3 protein levels were unchanged in the mutant cell lines (Fig 3A), we were surprised to observe downregulation of SMC3 at the gene level, as well as two of the other three members of the cohesin ring (Rad21 and STAG2), in 8CDm cells compared to WT. Conversely, SMC3 gene expression was up-regulated in 8KO cells, as were SMC1A and STAG2 to a lesser magnitude (Fig 5). This striking difference between the cell lines suggested a major difference in what was occurring in cells containing a catalytically-inactive version of KDAC8 (8CDm), compared to cells in which KDAC8 was completely missing (8KO).

**Fig 5:**
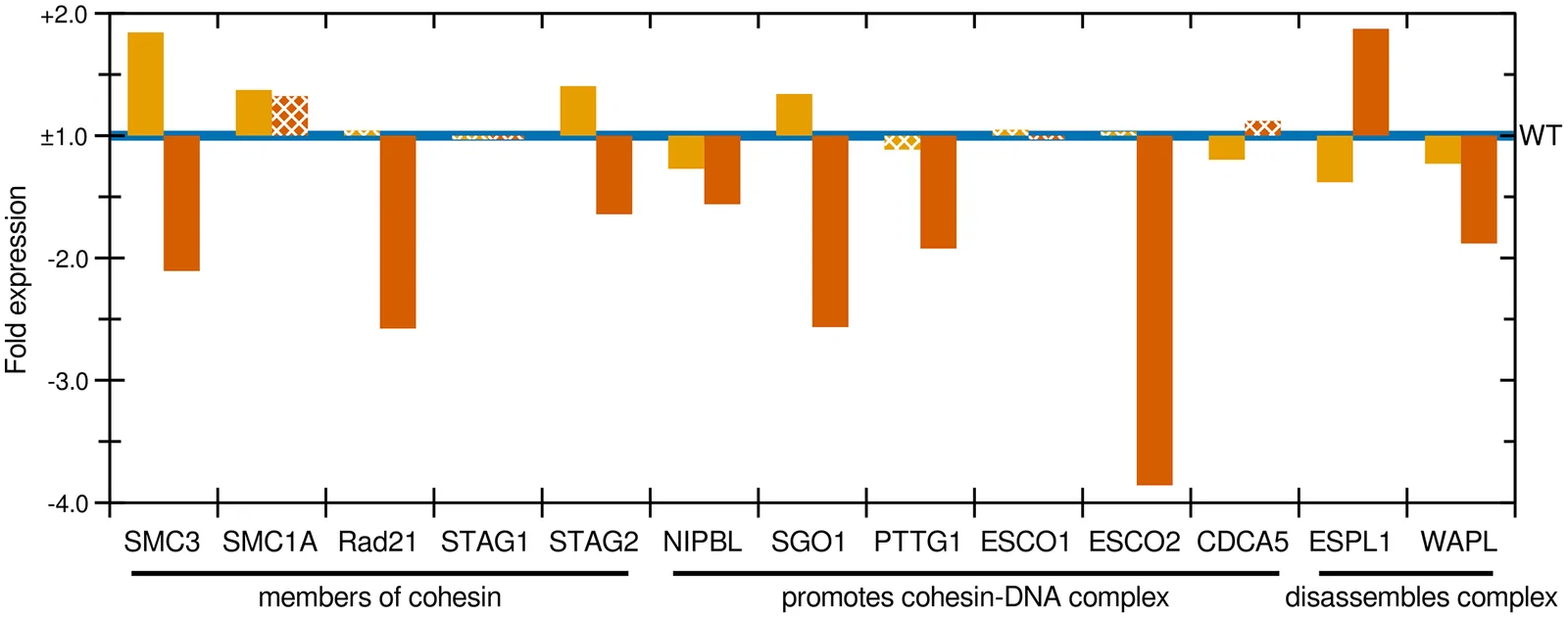
8CDm and 8KO cells differentially regulate cohesin-related genes. RNA-seq gene expression levels for cohesin-related genes. Fold expression compared to WT in 8KO (yellow) and 8CDm (red) cells are plotted for each gene. WT expression (blue line) has been set to 1 for each gene. Significant changes (false discovery rate < 0.001) are represented by solid bars, while hatched bars are not significantly different from WT.

Next, we examined mRNA expression of proteins that promote binding of cohesin to DNA and the proteins driving release of cohesin from DNA. Several proteins that contribute to formation and stabilization of the cohesin complex on DNA, especially for sister chromatid cohesion, were also downregulated in 8CDm cells at the mRNA level. These included ESCO2 (the acetyltransferase primarily responsible for acetylating SMC3 for sister chromatid cohesion) and NIPBL (responsible for loading cohesin onto DNA), as well as other binding proteins responsible for stabilizing the cohesin-DNA complex (Fig 5). When considering gene expression of proteins critical in removing cohesin from DNA, the trend is less clear. In 8CDm cells, WAPL is downregulated while ESPL1 (separase) is upregulated. These two proteins represent two alternative pathways for removing the cohesin ring from DNA, the latter of which is primarily utilized during sister chromatid cohesion and segregation due to the more stable chromatin-DNA complex. In the 8KO cell line, the expression changes for cohesin-associated proteins were both generally smaller in magnitude and did not show a discernable trend in the directionality of the expression changes (Fig 5). The gene expression data reinforced that the presence of 8CDm caused a more dramatic perturbation of cell cycle-related processes than loss of KDAC8 in the cells.

## Discussion

The approach taken in this study has confirmed CIN as a phenotype associated with the presence of inactive KDAC8 (Figs 3 and 4), consistent with the prior observations in cells from CdLS patients over a shorter time span and effects of altered SMC3 acetylation [25,26]. Overall, these data indicate that KDAC8 plays a role in maintaining chromosome stability, which is likely mediated through its previously characterized SMC3 deacetylation function [15,23]. The generation of a stable cell line resulting in expression of either 8CDm or 8KO from the endogenous locus occurred over several dozen generations. The 8KO and 8CDm cell lines represent the long-term adapted state upon loss of KDAC8 or presence of a catalytically-inactive KDAC8, respectively. Phenotypic differences observed between the cell lines are determined by the cells adaptive response to these two different mutations over time. Thus, our analysis revealed the accumulated response to the continuous presence of 8CDm and loss of KDAC8 catalytic activity, including decreased expression of several key genes involved in this pathway and the accumulation of genomic aberrations far past what transient transfection or knock-down experiments would allow.

It is also plausible that the cancerous nature of HT1080 cells exacerbated the observed phenotypes, facilitating the discovery of this role for KDAC8, as these cells likely tolerate more CIN than many other cell types. Nevertheless, our observations are consistent with previous data, as genome duplication and CIN have been observed, albeit less severely, upon knockdown of other cohesin-related proteins and manipulation of ESCO1 and ESCO2 leading to altered SMC3 acetylation [16,26]. Interestingly, the authors of the latter study reported that a KDAC8 knockout did not lead to an increase in micronuclei, also consistent with our observations [26]. Furthermore, prior work has shown reproducible induction of genomic instability through CRISPR/Cas9 inactivation of target proteins involved in the cell cycle without reported genomic instability from off-target double-strand breaks [34,35]. The 8KO cell line was generated using the same guide RNA as the 8CDm (i.e., an identical workflow except for lack of repair template), and none of the several pooled 8KO lines show the same defects as 8CDm, further suggesting that the 8CDm effects are specific to KDAC8 inactivation rather than an off-target effect. Altogether, the consistency of our results with prior observations strongly suggests that the CIN observed in the 8CDm line is due to disruption of the acetylation cycle of SMC3 caused by the presence of catalytically inactive KDAC8.

Even though the amount of acSMC3 was greatly increased in the mutant cells, total SMC3 levels remained constant (Fig 2A). Consistent with our data, constant total SMC3 levels have previously been observed in cells when KDAC8 or ESCO1/2 activity have been altered and increased acSMC3 observed [7,23,31,36,37]. We therefore conclude that the cells are actively maintaining a constant level of total SMC3, leading to an increase in the ratio of acetylated to unacetylated SMC3 in both mutant cell lines (Fig 2C). However, the 8CDm cell line has a greatly enhanced phenotype compared to the 8KO cell line, despite both lines having a drastic increase in the amount of acSMC3. We observed an increase in gene-level expression of SMC3 and other cohesin complex proteins in 8KO cells, but a decrease in gene-level expression of SMC3 and related proteins in 8CDm cells, which correlated to the more severe phenotype (Fig 5). Given that total SMC3 protein levels were the same despite the change in gene expression levels in our mutant cell lines, we reason that there is likely a compensatory change in protein degradation to account for our observations of constant SMC3 despite a net 4-fold difference in detected SMC3 mRNA between the 8CDm and 8KO cell lines. Several lines of evidence indicate that the half-life of SMC3 is near the median of the range for all proteins and long-lived compared to many cell cycle mediators (i.e., persistence for at least several hours), which supports a model in which usual turnover mechanisms of expression and degradation function to control the steady-state level of SMC3 [38–42]. Thus, the rate of SMC3 turnover may have increased in 8KO cells and decreased in 8CDm cells. It is technically possible that the cells could be compensating for the changes in SMC3 transcription levels by modulating the mRNA half-life or the translation rate to keep SMC3 levels steady in the mutant cell lines. However, it is unclear how these feedback loops would occur or how the presence of the 8CDm protein would lead to higher levels of mRNA degradation or lower levels of translation for specific proteins. Furthermore, this reasoning would also require an alternative, unidentified, mechanism in the 8KO cell line that could to produce a CIN phenotype similar to WT even though SMC3 is hyperacetylated. In cells in an unperturbed environment, consistent with our adapted cell lines, mRNA-level expression is highly correlated in direction with resulting protein expression [43]. Therefore, although we have not definitely ruled out some contribution from translation-level regulation in this process, it appears most probable that protein expression (and therefore degradation) rates have been altered in the same direction as the gene expression changes.

To understand how this proposed change in protein expression and degradation would lead to the difference in CIN phenotype in the two mutant cell lines, the acetylation cycle of SMC3 must be considered (Fig 1). In cells, SMC3 is either chromatin-bound as part of a cohesin complex or free. After complex assembly and chromatin-binding, some fraction of chromatin-bound SMC3 is acetylated. AcSMC3 is eventually released from chromatin and is usually deacetylated by KDAC8, after which the free SMC3 can be reassembled into new cohesin complexes. Importantly, only unacetylated SMC3 can assemble into new DNA-bound cohesin complexes [5,15,23,44]. Neither the relative amounts of unacetylated SMC3 and acSMC3 in the cell nor the relative amounts of free vs. chromatin-bound SMC3 are known, in either mitotic or interphase cells; however, a constant amount of total SMC3 is apparently maintained by balancing expression and degradation levels, while existing SMC3 moves between these four states. In the 8KO and 8CDm cell lines, there is a higher ratio of acSMC3:SMC3, and the acSMC3 is not deacetylated. So when acSMC3 releases from chromatin, it must accumulate in the free state until it is degraded, as only unacetylated SMC3 has been reported to form new complexes [5,15,23,44]. In the absence of active KDAC8 (and assuming there is not a completely unknown alternative process by which acSMC3 can otherwise be recycled), the accumulating acSMC3 must be degraded over time. The 8KO cells have upregulated SMC3 gene expression, presumably to produce fresh SMC3 to compensate for the inability to recycle acSMC3; given an increased production of SMC3 but a constant total amount of SMC3, the protein degradation rate of free SMC3 and/or acSMC3 must have correspondingly increased. In contrast, the 8CDm cells downregulated SMC3 gene expression; in order to maintain a constant SMC3 level, these cells have therefore reduced the rate of degradation of free SMC3 and/or acSMC3. Based on the rather mild phenotypes associated with loss of KDAC8 in the 8KO line, the compensatory mechanism of adjusting turnover rate is sufficient to ensure that there is enough SMC3 to assemble into chromatin-bound complexes for cohesin to function. In the 8CDm cells, the combination of accumulating unusable acSMC3 and reduced expression of SMC3 leads to an insufficient pool of free, unacetylated SMC3 for use in complex assembly and ultimately to the observed severe defects in this cell line. Although we have not directly probed the turnover of SMC3 in this study, the prior work establishing the relatively slow turnover of SMC3 compared to most cell cycle regulators has demonstrated that measuring the turnover of chromatin-bound SMC3 is not amenable to probing with standard techniques such as cycloheximide inhibition [39–42]. Slow turnover of SMC3 is consistent with the model that we have proposed here.

Our data do not directly address why the presence of 8CDm would result in the more severe phenotype or the decrease in SMC3 expression we have observed in the 8CDm cell line, but a straightforward possible mechanism can be postulated. 8CDm contains an inactivating mutation in a residue that is in the active site and is required for catalytic turnover (H143A), but is only slightly impaired for substrate binding and retains wild-type structure *in vitro* [45–47]. We propose that 8CDm, which can bind to acSMC3 but cannot catalyze a deacetylation reaction, is sequestering acSMC3 and somehow preventing its degradation in these cells, perhaps simply by blocking access to a key residue for initiation of degradation. To maintain the observed steady-state SMC3 levels in our hypothesized model, cells must therefore reduce expression of new SMC3. This simple model is not contingent on whether free acSMC3 is degraded through the same pathway as free SMC3 or via a separate pathway.

Together, these results and working model fit into existing models of KDAC8 function and contribute to a more comprehensive working model to explain how mutations in KDAC8, especially missense mutations which impair catalytic activity, drive an increase in CIN and may contribute to disease states (Fig 6). If 8KO cells entirely lacking KDAC8 can compensate for the inability to recycle acSMC3 into new cohesin complexes by up-regulating SMC3 and increasing protein turnover, the effects of altered SMC3 acetylation on chromosomal instability will be minimized. In 8CDm cells, the presence of defective KDAC8 that may sequester acSMC3 would therefore impair degradation of unusuable acSMC3, driving downregulation of the SMC3 gene to maintain a constant total SMC3 concentration. Reduced expression of new SMC3, as well as other members of the cohesin complex, combined with the inability to recycle previously acetylated SMC3, would therefore result in a loss of functional cohesin complexes. Ultimately, the shortage of cohesin complexes over several rounds of cell division would lead to issues with proper chromosome segregation and CIN. While this proposal is consistent with the current understanding of the role and mechanisms of SMC3 in cohesin, verification of specific mechanistic details of this model, particularly with respect to SMC3 turnover and free vs. bound populations, need to be explored in future work to validate the general applicability of the model.

**Fig 6:**
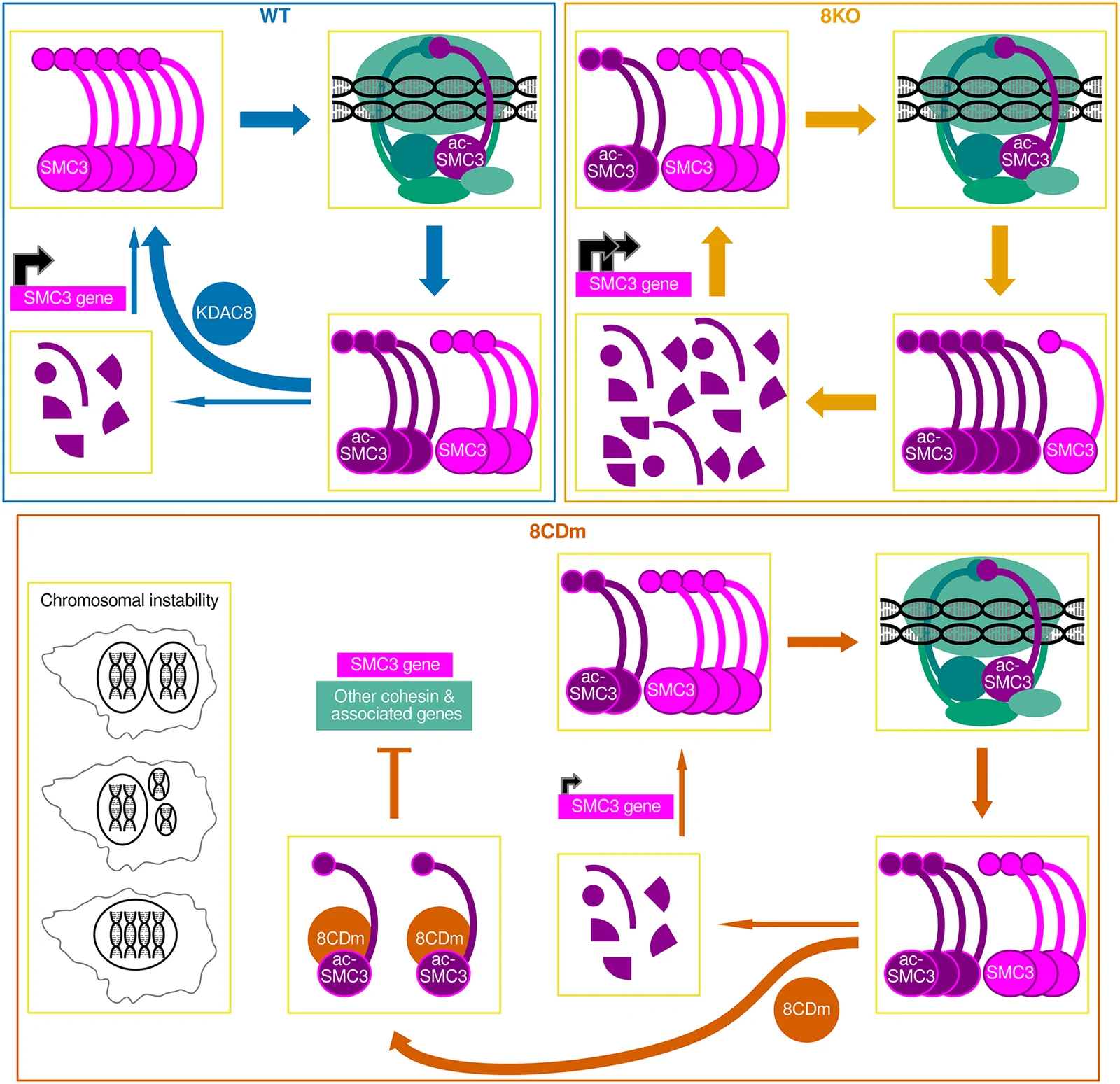
Cellular outcomes upon chronic loss of KDAC8 activity. Schematic illustrating hypothesized differences in cohesin recycling and turnover throughout a cell cycle in WT (blue box), 8KO (yellow box), and 8CDm (red box) cell lines that are consistent with the observed phenotype outcomes. WT cells can effectively recycle SMC3 for incorporation into new cohesin complexes through deacetylation, while also maintaining a steady state level of SMC3 protein turnover. Both 8KO and 8CDm cell lines maintain a higher ratio of acSMC3 to SMC3 than WT cells, as SMC3 is not able to be deacetylated (and therefore not re-incorporated into cohesin complexes) in these lines. Data presented here suggest a compensatory mechanism in the absence of KDAC8 (8KO), including increased SMC3 degradation and expression for subsequent cell cycles. Inactive KDAC8 (8CDm) may bind acSMC3 after cohesin disassociation, leading to reduced SMC3 turnover as well as decreased expression of new cohesin components and complex-promoting proteins. Lack of SMC3 and cohesin-associated proteins over several cell cycles would then lead to chromosomal instability in 8CDm cells. Thinner arrows represent paths hypothesized to be followed with lower frequency compared to thicker arrows.

We recognize that it is also possible that KDAC8 has an unidentified deacetylation or binding target in cells that may be driving or contributing to the observed phenotypes. ARID1A is another putative target of KDAC8 that could plausibly contribute to chromatin instability, as it is a core member of a subset of SWI/SNF chromatin remodeling complexes [48]. However, the evidence for deacetylation of ARID1A by KDAC8 primarily comes from *in vitro* peptide-based assays, and cell-based assays have not produced a consistent association between the two proteins [32,49–51]. Moreover, disruption of KDAC8 and ARID1A functions appear to lead to distinct and clearly distinguishable disease states, suggesting that deacetylation of ARID1A is not likely to be the primary driver of our observed phenotypes [52]. Nearly all putative substrates of KDAC8 have been suggested based on dosing of cells with the KDAC inhibitor PCI-34051 (including ARID1A), which has been demonstrated to have significant off-target effects that potentially invalidate any association of altered phenotypes with KDAC8, or with even less cell-based evidence than for ARID1A [28,29,31,53]. Therefore, we have focused our analysis here solely on SMC3, as it is the only well-validated target of KDAC8 and has known consequences of disruption that are consistent with all of our observed phenotypes. We acknowledge that future work may associate some of our observed outcomes with contributions from interactions of KDAC8 with proteins not yet confirmed or suspected as targets.

Our overall observation that presence of the inactive KDAC8 exacerbates the CIN phenotype in HT1080 cells suggests why missense mutations in KDAC8 drive a wide range of severity in CdLS patients, and in some cases more severe disease than complete loss of the protein [22]. 8CDm is analogous to many CdLS-causative KDAC8 variants with reduced or no measurable catalytic activity. Our results suggest that a mechanism resulting in alteration of SMC3 turnover and cohesin complex abundance may contribute to disease severity providing an explanation for why some KDAC8 mutations may lead to more or less severe outcomes in CdLS patients compared to patients with mutations resulting in elimination of KDAC8 expression. Although acetylation of SMC3 seems to be less critical during interphase than during mitosis, accumulation of acSMC3 in the absence of functional KDAC8 leads to a reduction in free unacetylated SMC3 available to form *de novo* cohesin complexes. Ultimately, less free SMC3 would lead to less chromatin-associated cohesin and would be problematic for gene regulation. Even in non-cancerous cells that would be unlikely to accumulate the extensive CIN that we observed in HT1080 cells, the impacts on SMC3 turnover would persist, disrupting interphase functions of the cohesin complex. The current majority view is that CdLS is a consequence of cohesin-mediated gene regulation defects rather than CIN [4,22,23,25]. However our results and the reported observations of CIN in cells of CdLS patients suggest that CIN may also be a significant factor in disease progression [25].

Further investigation into what is driving the disparate phenotypes in these two cell lines will undoubtedly shed additional insight on the role of KDAC8 in cells, especially correlating protein expression with mRNA expression and probing turnover rates of SMC3. Another important question is whether KDAC8 has other critical substrates in addition to SMC3 which cannot otherwise be deacetylated in the absence of KDAC8 activity. Our additions to the model of the acetylation cycle of the cohesin complex extend our understanding of potential variation in cohesinopathies and the relative importance of SMC3 acetylation in mitotic versus interphase processes.

## Supporting information

S1 Fig Validation of 8KO cell line. Chromatograms resulting from Saenger sequencing of PCR from genomic DNA encompassing the region in which a mutation was expected. Translations are shown under chromatogram using one-letter amino acid code. Mutation sites are shown for all 8KO isolates as black arrow. Four independent strains isolated after single-cell dilution resulted in a frameshift mutation which led to a stop codon after aa147. These strains were pooled to make the 8KO cell line used in all experiments reported here.
(PDF)

S2 Fig Full immunoblots. Full length immunoblots for data shown in Fig 2.
(PDF)

S3 Fig Volume distributions of 8CDm cells are consistent with cycling cells. Front scatter area data for individual cell populations in Fig 3B. Mean cell volume of WT cells containing 2n DNA (G1 phase) was set to 1. p values were determined by Mann-Whitney U tests.
(PDF)

S4 Fig All cell lines have similar progression through the cell cycle as assessed by live cell imaging. Snapshots of representative individual cell division events from each cell line taken from live cell phase imaging. For each series, the rounded cell was set at 0 min and the elapsed cell division time is noted under each image. The scale bar represents 5 µm.
(PDF)

S1 Table Statistical descriptors for all data series. (XLSX)

S2 Table Significant gene expression changes in 8KO relative to wild-type. (XLSX)

S3 Table Significant GO terms based on 8KO gene expression changes of false discovery rate < 0.001. (XLSX)

## References

[1] Mohanta TK, Mishra AK, Al-Harrasi A. The 3D genome: From structure to function. Int J Mol Sci. 2021;22(21):11585. doi: 10.3390/ijms222111585 34769016 PMC8584255

[2] Kim Y, Yu H. Shaping of the 3D genome by the ATPase machine cohesin. Exp Mol Med. 2020;52(12):1891–7. doi: 10.1038/s12276-020-00526-2 33268833 PMC8080590

[3] Di Nardo M, Musio A. Cohesin - bridging the gap among gene transcription, genome stability, and human diseases. FEBS Lett. 2025;599(2):190–208. doi: 10.1002/1873-3468.14949 38852996

[4] Solé-Ferran M, Losada A. Cohesin in 3D: Development, differentiation, and disease. Genes Dev. 2025;genesdev;gad.352671.125v1. doi: 10.1101/gad.352671.125 PMC12128871 40345853

[5] Beckouët F, Hu B, Roig MB, Sutani T, Komata M, Uluocak P, et al. An Smc3 acetylation cycle is essential for establishment of sister chromatid cohesion. Mol Cell. 2010;39(5):689–99. doi: 10.1016/j.molcel.2010.08.008 20832721 PMC4766734

[6] Zhang J, Shi X, Li Y, Kim B-J, Jia J, Huang Z, et al. Acetylation of Smc3 by Eco1 is required for S phase sister chromatid cohesion in both human and yeast. Mol Cell. 2008;31(1):143–51. doi: 10.1016/j.molcel.2008.06.006 18614053

[7] Alomer RM, da Silva EML, Chen J, Piekarz KM, McDonald K, Sansam CG, et al. Esco1 and Esco2 regulate distinct cohesin functions during cell cycle progression. Proc Natl Acad Sci U S A. 2017;114(37):9906–11. doi: 10.1073/pnas.1708291114 28847955 PMC5604028

[8] Kawasumi R, Abe T, Arakawa H, Garre M, Hirota K, Branzei D. ESCO1/2’s roles in chromosome structure and interphase chromatin organization. Genes Dev. 2017;31(21):2136–50. doi: 10.1101/gad.306084.117 29196537 PMC5749162

[9] Gerlich D, Koch B, Dupeux F, Peters J-M, Ellenberg J. Live-cell imaging reveals a stable cohesin-chromatin interaction after but not before DNA replication. Curr Biol. 2006;16(15):1571–8. doi: 10.1016/j.cub.2006.06.068 16890534

[10] Rankin S, Ayad NG, Kirschner MW. Sororin, a substrate of the anaphase-promoting complex, is required for sister chromatid cohesion in vertebrates. Mol Cell. 2005;18(2):185–200. doi: 10.1016/j.molcel.2005.03.017 15837422

[11] Nishiyama T, Ladurner R, Schmitz J, Kreidl E, Schleiffer A, Bhaskara V, et al. Sororin mediates sister chromatid cohesion by antagonizing Wapl. Cell. 2010;143(5):737–49. doi: 10.1016/j.cell.2010.10.031 21111234

[12] McGuinness BE, Hirota T, Kudo NR, Peters J-M, Nasmyth K. Shugoshin prevents dissociation of cohesin from centromeres during mitosis in vertebrate cells. PLoS Biology. 2005;3:e86. doi: 10.1371/journal.pbio.0030086 PMC1054882 15737064

[13] Uhlmann F, Wernic D, Poupart MA, Koonin EV, Nasmyth K. Cleavage of cohesin by the CD clan protease separin triggers anaphase in yeast. Cell. 2000;103(3):375–86. doi: 10.1016/s0092-8674(00)00130-6 11081625

[14] Ciosk R, Zachariae W, Michaelis C, Shevchenko A, Mann M, Nasmyth K. An ESP1/PDS1 complex regulates loss of sister chromatid cohesion at the metaphase to anaphase transition in yeast. Cell. 1998;93(6):1067–76. doi: 10.1016/s0092-8674(00)81211-8 9635435

[15] Borges V, Lehane C, Lopez-Serra L, Flynn H, Skehel M, Rolef Ben-Shahar T, et al. Hos1 deacetylates Smc3 to close the cohesin acetylation cycle. Mol Cell. 2010;39(5):677–88. doi: 10.1016/j.molcel.2010.08.009 20832720

[16] Barber TD, McManus K, Yuen KWY, Reis M, Parmigiani G, Shen D, et al. Chromatid cohesion defects may underlie chromosome instability in human colorectal cancers. Proc Natl Acad Sci U S A. 2008;105(9):3443–8. doi: 10.1073/pnas.0712384105 18299561 PMC2265152

[17] Kirsch-Volders M, Mišík M, Fenech M. Tetraploidy in normal tissues and diseases: Mechanisms and consequences. Chromosoma. 2025;134(1):3. doi: 10.1007/s00412-025-00829-1 40117022 PMC11928420

[18] Guscott M, Saha A, Maharaj J, McClelland SE. The multifaceted role of micronuclei in tumour progression: A whole organism perspective. Int J Biochem Cell Biol. 2022;152:106300. doi: 10.1016/j.biocel.2022.106300 36189461

[19] Garribba L, Santaguida S. The dynamic instability of the aneuploid genome. Front Cell Dev Biol. 2022;10:838928. doi: 10.3389/fcell.2022.838928 35265623 PMC8899291

[20] Hosea R, Hillary S, Naqvi S, Wu S, Kasim V. The two sides of chromosomal instability: Drivers and brakes in cancer. Signal Transduct Target Ther. 2024;9(1):75. doi: 10.1038/s41392-024-01767-7 38553459 PMC10980778

[21] Kaur M, Blair J, Devkota B, Fortunato S, Clark D, Lawrence A, et al. Genomic analyses in Cornelia de Lange Syndrome and related diagnoses: Novel candidate genes, genotype-phenotype correlations and common mechanisms. Am J Med Genet A. 2023;191(8):2113–31. doi: 10.1002/ajmg.a.63247 37377026 PMC10524367

[22] Kaiser FJ, Ansari M, Braunholz D, Concepción Gil-Rodríguez M, Decroos C, Wilde JJ, et al. Loss-of-function HDAC8 mutations cause a phenotypic spectrum of Cornelia de Lange syndrome-like features, ocular hypertelorism, large fontanelle and X-linked inheritance. Hum Mol Genet. 2014;23(11):2888–900. doi: 10.1093/hmg/ddu002 24403048 PMC4014191

[23] Deardorff MA, Bando M, Nakato R, Watrin E, Itoh T, Minamino M, et al. HDAC8 mutations in Cornelia de Lange syndrome affect the cohesin acetylation cycle. Nature. 2012;489(7415):313–7. doi: 10.1038/nature11316 22885700 PMC3443318

[24] Haberland M, Mokalled MH, Montgomery RL, Olson EN. Epigenetic control of skull morphogenesis by histone deacetylase 8. Genes Dev. 2009;23(14):1625–30. doi: 10.1101/gad.1809209 19605684 PMC2714711

[25] Di Nardo M, Krantz ID, Musio A. Genome instability and senescence are markers of Cornelia de Lange Syndrome cells. Cells. 2024;13(23):2025. doi: 10.3390/cells13232025 39682772 PMC11640591

[26] Adams DJ, Barlas B, McIntyre RE, Salguero I, van der Weyden L, Barros A, et al. Genetic determinants of micronucleus formation in vivo. Nature. 2024;627(8002):130–6. doi: 10.1038/s41586-023-07009-0 38355793 PMC10917660

[27] Balasubramanian S, Ramos J, Luo W, Sirisawad M, Verner E, Buggy JJ. A novel histone deacetylase 8 (HDAC8)-specific inhibitor PCI-34051 induces apoptosis in T-cell lymphomas. Leukemia. 2008;22(5):1026–34. doi: 10.1038/leu.2008.9 18256683

[28] Lechner S, Malgapo MIP, Grätz C, Steimbach RR, Baron A, Rüther P, et al. Target deconvolution of HDAC pharmacopoeia reveals MBLAC2 as common off-target. Nat Chem Biol. 2022;18(8):812–20. doi: 10.1038/s41589-022-01015-5 35484434 PMC9339481

[29] Bornes KE, Moody MA, Huckaba TM, Benz MC, McConnell EC, Foroozesh M, et al. Lysine deacetylase inhibitors have low selectivity in cells and exhibit predominantly off-target effects. FEBS Open Bio. 2025;15(1):94–107. doi: 10.1002/2211-5463.13896 39482806 PMC11705486

[30] Gantt SL, Gattis SG, Fierke CA. Catalytic activity and inhibition of human histone deacetylase 8 is dependent on the identity of the active site metal ion. Biochemistry. 2006;45(19):6170–8. doi: 10.1021/bi060212u 16681389

[31] Toro TB, Skripnikova EV, Bornes KE, Zhang K, Watt TJ. Endogenous expression of inactive lysine deacetylases reveals deacetylation-dependent cellular mechanisms. PLoS One. 2023;18(9):e0291779. doi: 10.1371/journal.pone.0291779 37721967 PMC10506724

[32] Toro TB, Watt TJ. Critical review of non-histone human substrates of metal-dependent lysine deacetylases. FASEB J. 2020;34(10):13140–55. doi: 10.1096/fj.202001301RR 32862458 PMC7719617

[33] Meijering E, Dzyubachyk O, Smal I. Methods for cell and particle tracking. Methods Enzymol. 2012;504:183–200. doi: 10.1016/B978-0-12-391857-4.00009-4 22264535

[34] Banaszak LG, Giudice V, Zhao X, Wu Z, Gao S, Hosokawa K, et al. Abnormal RNA splicing and genomic instability after induction of DNMT3A mutations by CRISPR/Cas9 gene editing. Blood Cells Mol Dis. 2018;69:10–22. doi: 10.1016/j.bcmd.2017.12.002 29324392 PMC6728079

[35] Panagopoulos A, Stout M, Kilic S, Leary P, Vornberger J, Pasti V, et al. Multigenerational cell tracking of DNA replication and heritable DNA damage. Nature. 2025;642(8068):785–95. doi: 10.1038/s41586-025-08986-0 40399682 PMC12176655

[36] van Ruiten MS, van Gent D, Sedeño Cacciatore Á, Fauster A, Willems L, Hekkelman ML, et al. The cohesin acetylation cycle controls chromatin loop length through a PDS5A brake mechanism. Nat Struct Mol Biol. 2022;29(6):586–91. doi: 10.1038/s41594-022-00773-z 35710836 PMC9205776

[37] Sang C, Li X, Liu J, Chen Z, Xia M, Yu M, et al. Reversible acetylation of HDAC8 regulates cell cycle. EMBO Rep. 2024;25(9):3925–43. doi: 10.1038/s44319-024-00210-w 39043961 PMC11387496

[38] Christiano R, Nagaraj N, Fröhlich F, Walther TC. Global proteome turnover analyses of the Yeasts S. cerevisiae and S. pombe. Cell Rep. 2014;9(5):1959–65. doi: 10.1016/j.celrep.2014.10.065 25466257 PMC4526151

[39] Orgil O, Mor H, Matityahu A, Onn I. Identification of a region in the coiled-coil domain of Smc3 that is essential for cohesin activity. Nucleic Acids Res. 2016;44(13):6309–17. doi: 10.1093/nar/gkw539 27307603 PMC5291275

[40] Kulemzina I, Schumacher MR, Verma V, Reiter J, Metzler J, Failla AV, et al. Cohesin rings devoid of Scc3 and Pds5 maintain their stable association with the DNA. PLoS Genet. 2012;8(8):e1002856. doi: 10.1371/journal.pgen.1002856 22912589 PMC3415457

[41] Laugsch M, Seebach J, Schnittler H, Jessberger R. Imbalance of SMC1 and SMC3 cohesins causes specific and distinct effects. PLoS One. 2013;8(6):e65149. doi: 10.1371/journal.pone.0065149 23776448 PMC3680458

[42] Rega C, Tsitsa I, Roumeliotis TI, Krystkowiak I, Portillo M, Yu L, et al. High resolution profiling of cell cycle-dependent protein and phosphorylation abundance changes in non-transformed cells. Nat Commun. 2025;16(1):2579. doi: 10.1038/s41467-025-57537-8 40089461 PMC11910661

[43] Liu Y, Beyer A, Aebersold R. On the dependency of cellular protein levels on mRNA abundance. Cell. 2016;165(3):535–50. doi: 10.1016/j.cell.2016.03.014 27104977

[44] Xiong B, Lu S, Gerton JL. Hos1 is a lysine deacetylase for the Smc3 subunit of cohesin. Curr Biol. 2010;20(18):1660–5. doi: 10.1016/j.cub.2010.08.019 20797861

[45] Gantt SML, Decroos C, Lee MS, Gullett LE, Bowman CM, Christianson DW, et al. General base-general acid catalysis in human histone deacetylase 8. Biochemistry. 2016;55(5):820–32. doi: 10.1021/acs.biochem.5b01327 26806311 PMC4747815

[46] Dowling DP, Gantt SL, Gattis SG, Fierke CA, Christianson DW. Structural studies of human histone deacetylase 8 and its site-specific variants complexed with substrate and inhibitors. Biochemistry. 2008;47(51):13554–63. doi: 10.1021/bi801610c 19053282 PMC2635894

[47] Toro TB, Painter RG, Haynes RA, Glotser EY, Bratton MR, Bryant JR, et al. Purification of metal-dependent lysine deacetylases with consistently high activity. Protein Expr Purif. 2018;141:1–6. doi: 10.1016/j.pep.2017.08.009 28843507 PMC5624855

[48] Mashtalir N, D’Avino AR, Michel BC, Luo J, Pan J, Otto JE, et al. Modular organization and assembly of SWI/SNF family chromatin remodeling complexes. Cell. 2018;175(5):1272-1288.e20. doi: 10.1016/j.cell.2018.09.032 30343899 PMC6791824

[49] Toro TB, Watt TJ. KDAC8 substrate specificity quantified by a biologically relevant, label-free deacetylation assay. Protein Sci. 2015;24(12):2020–32. doi: 10.1002/pro.2813 26402585 PMC4815229

[50] Olson DE, Udeshi ND, Wolfson NA, Pitcairn CA, Sullivan ED, Jaffe JD, et al. An unbiased approach to identify endogenous substrates of “histone” deacetylase 8. ACS Chem Biol. 2014;9(10):2210–6. doi: 10.1021/cb500492r 25089360 PMC4201337

[51] Lopez JE, Haynes SE, Majmudar JD, Martin BR, Fierke CA. HDAC8 substrates identified by genetically encoded active site photocrosslinking. J Am Chem Soc. 2017;139(45):16222–7. doi: 10.1021/jacs.7b07603 29035536 PMC5865639

[52] Dyment DA, O’Donnell-Luria A, Agrawal PB, Coban Akdemir Z, Aleck KA, Antaki D, et al. Alternative genomic diagnoses for individuals with a clinical diagnosis of Dubowitz syndrome. Am J Med Genet A. 2021;185(1):119–33. doi: 10.1002/ajmg.a.61926 33098347 PMC8197629

[53] Chang Y-C, Gnann C, Steimbach RR, Bayer FP, Lechner S, Sakhteman A, et al. Decrypting lysine deacetylase inhibitor action and protein modifications by dose-resolved proteomics. Cell Rep. 2024;43(6):114272. doi: 10.1016/j.celrep.2024.114272 38795348

