# Supplementary material for: Loss of cohesin deacetylation through KDAC8 inactivation leads to altered cohesin complex expression and chromosomal instability
Source: PLoS One. 2026 Oct 29;21(10):e0361705. doi: 10.1371/journal.pone.0361705 (PMC13718912; doi:10.1371/journal.pone.0361705)
Supplement: Supplementary file 1 [file pone.0361705.s001.pdf]

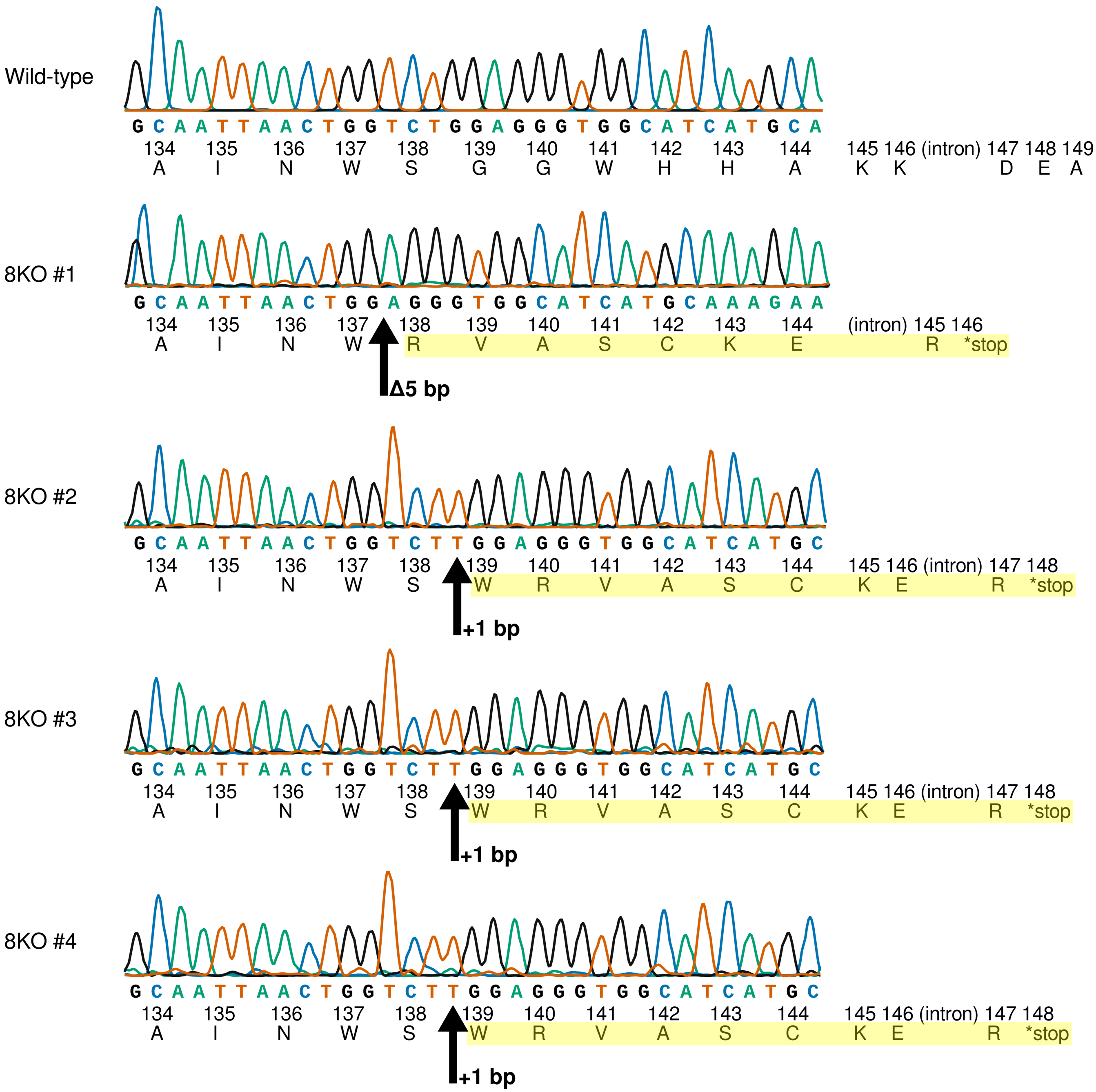

S1 Fig. Validation of 8KO cell line. Chromatograms resulting from Saenger sequencing of PCR from genomic DNA encompassing the region in which a mutation was expected. Translations are shown under chromatogram using one-letter amino acid code. Mutation sites are shown for all 8KO isolates as black arrow. Four independent strains isolated after single-cell dilution resulted in a frameshift mutation which led to a stop codon after aa147. These strains were pooled to make the 8KO cell line used in all experiments reported here.
