# Supplementary material for: Loss of cohesin deacetylation through KDAC8 inactivation leads to altered cohesin complex expression and chromosomal instability
Source: PLoS One. 2026 Oct 29;21(10):e0361705. doi: 10.1371/journal.pone.0361705 (PMC13718912; doi:10.1371/journal.pone.0361705)
Supplement: Supplementary file 2 [file pone.0361705.s002.pdf]

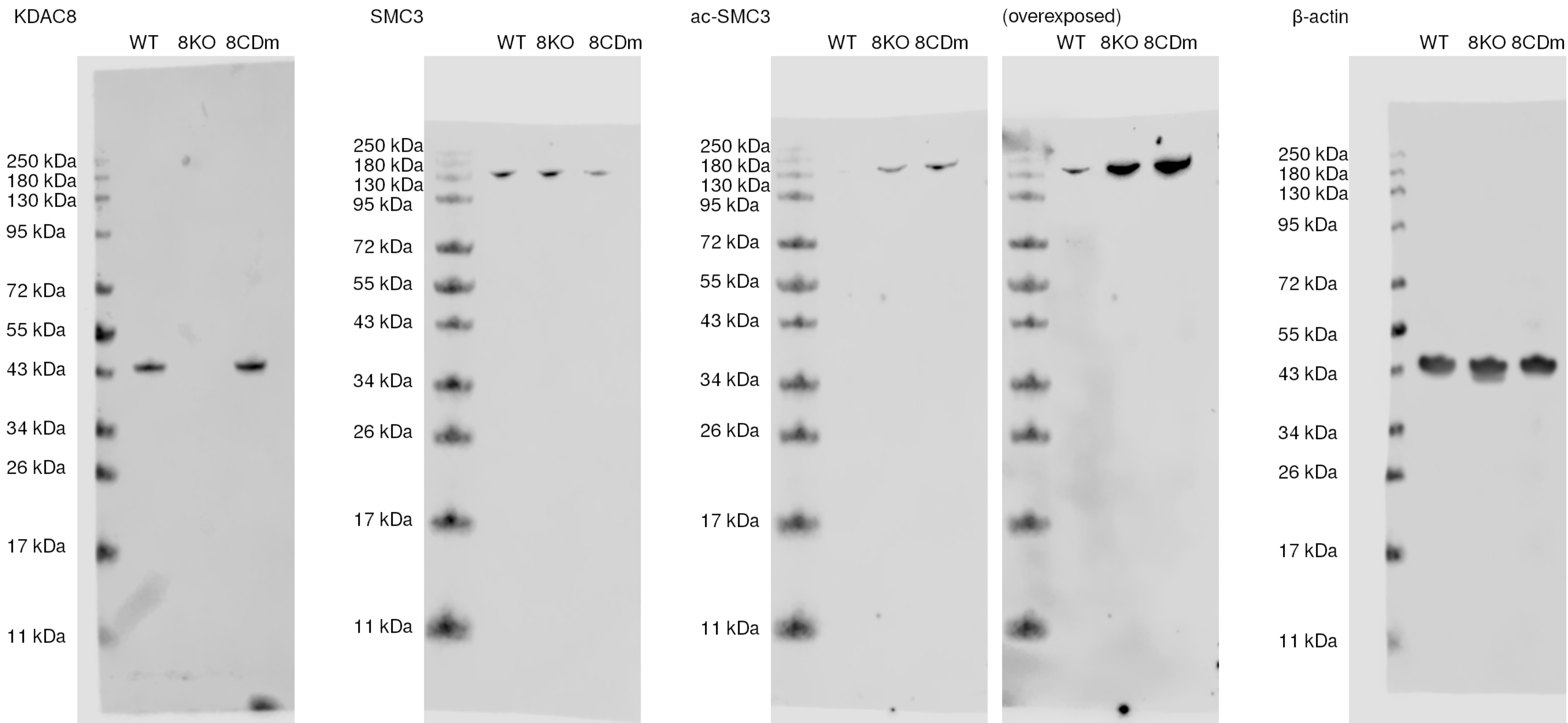

S2 Fig. Full western blots. Full length western blots for data shown in Figure 2.

Loss of cohesin deacetylation through KDAC8 inactivation leads to altered cohesin complex expression and chromosomal instability. Tasha B. Toro, Razan H. Hammad, Thomas M. Huckaba, Terry J. Watt.
