# Supplementary material for: Loss of cohesin deacetylation through KDAC8 inactivation leads to altered cohesin complex expression and chromosomal instability
Source: PLoS One. 2026 Oct 29;21(10):e0361705. doi: 10.1371/journal.pone.0361705 (PMC13718912; doi:10.1371/journal.pone.0361705)
Supplement: Supplementary file 3 [file pone.0361705.s003.pdf]

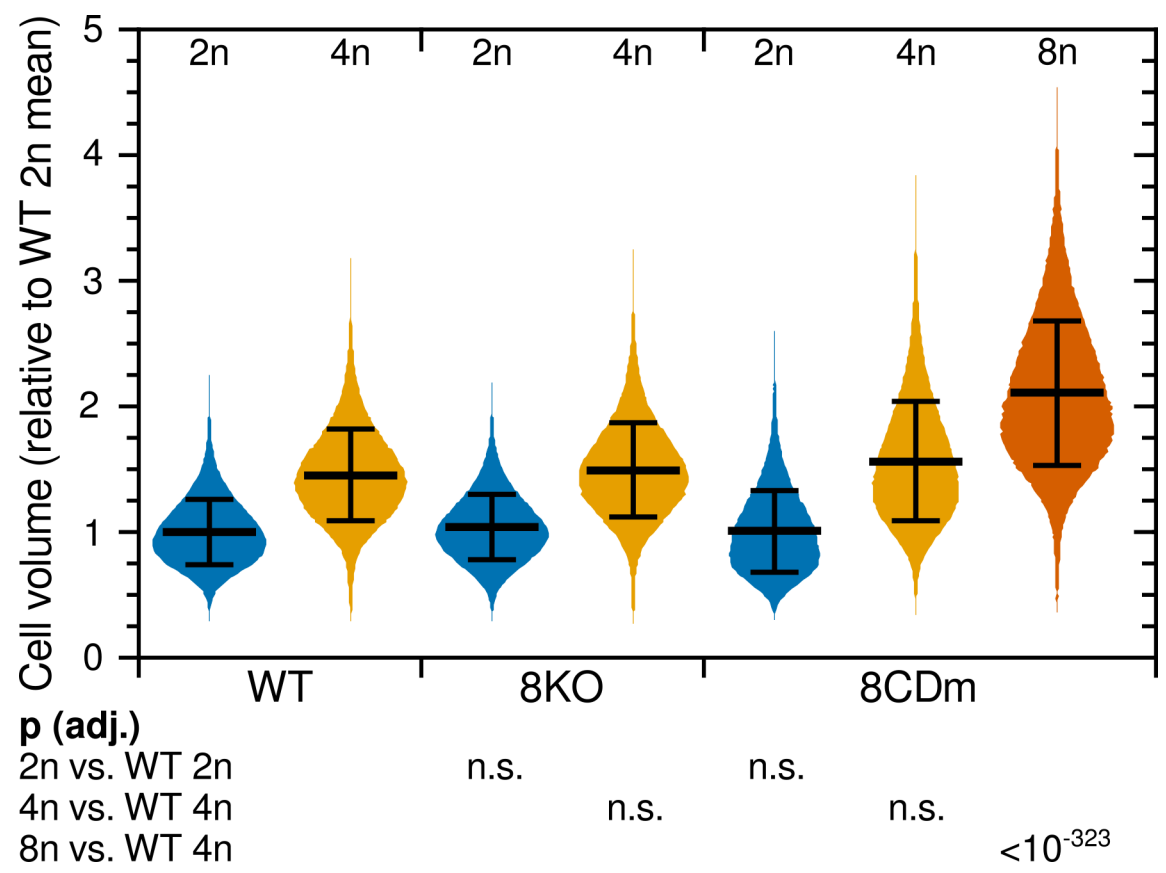

S3 Fig. Volume distributions of 8CDm cells are consistent with cycling cells. Front scatter area data for individual cell populations in Fig 3B. Mean cell volume of WT cells containing 2n DNA (G1 phase) was set to 1. p values were determined by Mann-Whitney U tests.
