# Supplementary material for: Loss of cohesin deacetylation through KDAC8 inactivation leads to altered cohesin complex expression and chromosomal instability
Source: PLoS One. 2026 Oct 29;21(10):e0361705. doi: 10.1371/journal.pone.0361705 (PMC13718912; doi:10.1371/journal.pone.0361705)
Supplement: Supplementary file 4 [file pone.0361705.s004.pdf]

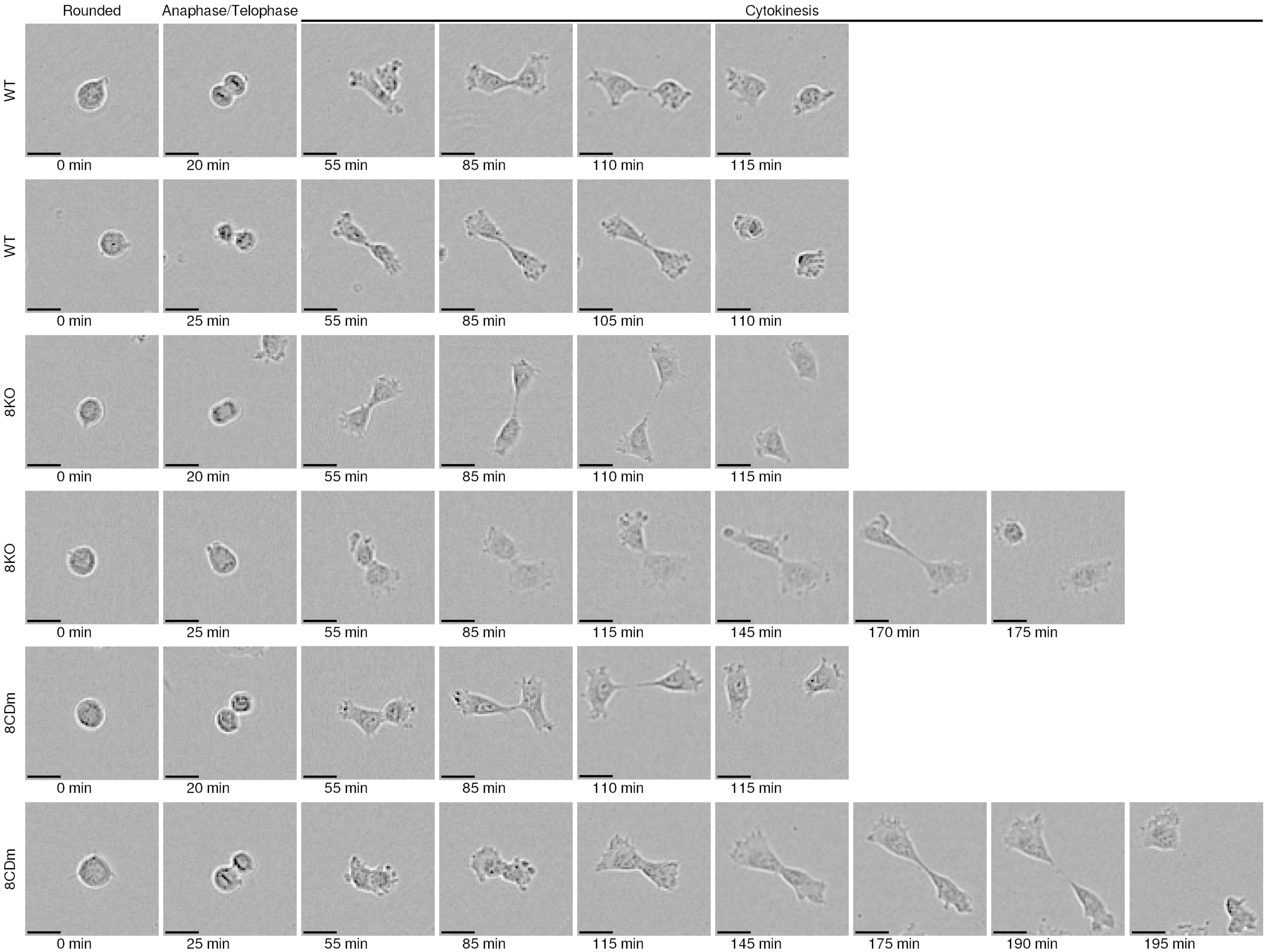

S4 Fig. All cell lines have similar progression through the cell cycle as assessed by live cell imaging. Snapshots of representative individual cell division events from each cell line taken from live cell phase imaging. For each series, the rounded cell was set at 0 min and the elapsed cell division time is noted under each image. The scale bar represents 5 µm.
